# Bis(1,3-dimesitylimidazol­yl)gold(I) 2,4,8,10-tetra­phenyl-1,3,5,7,9,11-hexa­oxa-2,4,8,10-tetra­bora-6-borataspiro­[5.5]undeca­ne

**DOI:** 10.1107/S1600536811016357

**Published:** 2011-05-07

**Authors:** Lennart Möhlmann, Ola F. Wendt, Magnus T. Johnson

**Affiliations:** a Centre for Analysis and Synthesis, Department of Chemistry, Lund University, PO Box 124, S-221 00 Lund, Sweden

## Abstract

The Au^I^ atom in the title compound, [Au(C_21_H_24_N_2_)_2_](C_24_H_20_B_5_O_6_), adopts a slightly distorted linear AuC_2_ coordination geometry arising from its coordination by two mesitylenic *N*-heterocyclic carbene ligands, forming an overall cationic complex. The dihedral angle between the imidazole rings is 57.3 (6)°. In the crystal, the components are linked by weak C—H⋯O hydrogen bonds.

## Related literature

For homoleptic bis-*N*-heterocyclic carbene complexes of gold(I), see, for example: Raubenheimer *et al.* (1996 ▶); Wang *et al.* (2005 ▶). For carbene complexes, see: Raubenheimer *et al.* (1996 ▶); Gaillard *et al.* (2010 ▶). For an overview of studies on a variety of bis-*N*-heterocyclic carbene complexes of gold(I) and their toxicity towards cancer cells, see: Teyssot *et al.* (2009 ▶) and for implications on mitochondrial directed chemoterapeutics, see: Baker *et al.* (2006 ▶); Barnard & Berners-Price (2007 ▶); Hickey *et al.* (2008 ▶). For a similar spiro tetra­aryl­penta­borate anion, see Nishihara *et al.* (2004 ▶). The title compound was obtained as a side product in the synthesis of the (1,3-dimesitylimidazolium)gold phenyl complex in an attempt to transmetallate the NHC gold(I) *tert*-butoxide (Johnson *et al.*, 2011) ▶ using phenyl boronic acid. A different approach leading to the NHC gold(I) phenyl complex was reported by Pazicky *et al.* (2010 ▶). For π–π inter­actions, see: Haddon (1982 ▶).
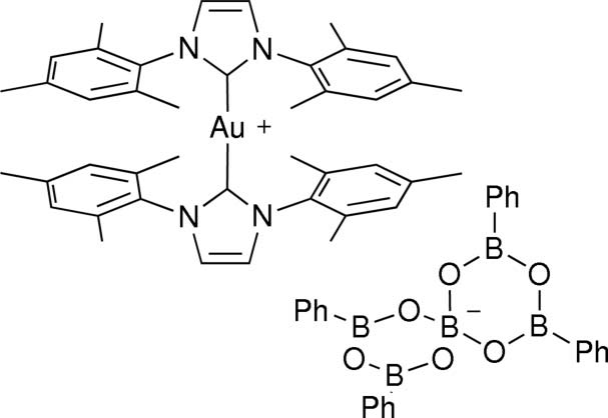


## Experimental

### 

#### Crystal data

[Au(C_21_H_24_N_2_)_2_](C_24_H_20_B_5_O_6_)
*M* *_r_* = 1264.26
Triclinic, 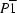

*a* = 15.388 (2) Å
*b* = 15.768 (2) Å
*c* = 16.453 (2) Å
α = 94.81 (1)°
β = 112.86 (1)°
γ = 117.65 (1)°
*V* = 3083.2 Å^3^
*Z* = 2
Mo *K*α radiation
μ = 2.44 mm^−1^
*T* = 293 K
0.20 × 0.20 × 0.10 mm

#### Data collection

Oxford Diffraction Xcalibur-3 CCD diffractometer
Absorption correction: multi-scan (*CrysAlis RED*; Oxford Diffraction, 2006 ▶) *T*_min_ = 0.838, *T*_max_ = 1.000
19866 measured reflections
10639 independent reflections
7217 reflections with *I* > 2σ(*I*)
*R*_int_ = 0.051

#### Refinement

*R*[*F* ^2^ > 2σ(*F* ^2^)] = 0.046
*wR*(*F* ^2^) = 0.152
*S* = 1.08
10639 reflections
751 parameters
H-atom parameters constrained
Δρ_max_ = 1.02 e Å^−3^
Δρ_min_ = −0.91 e Å^−3^

### 

Data collection: *CrysAlis CCD* (Oxford Diffraction, 2006 ▶); cell refinement: *CrysAlis RED* (Oxford Diffraction, 2006 ▶); data reduction: *CrysAlis RED*; program(s) used to solve structure: *SIR97* (Altomare *et al.*, 1999 ▶); program(s) used to refine structure: *SHELXL97* (Sheldrick, 2008 ▶); molecular graphics: *DIAMOND* (Brandenburg, 2006 ▶); software used to prepare material for publication: *SHELXL97*.

## Supplementary Material

Crystal structure: contains datablocks global. DOI: 10.1107/S1600536811016357/hb5850sup1.cif

Additional supplementary materials:  crystallographic information; 3D view; checkCIF report

## Figures and Tables

**Table:** 

Au1—C1	1.982 (7)
Au1—C22	1.990 (8)

**Table:** 

C1—Au1—C22	176.8 (3)

**Table 2:** Hydrogen-bond geometry (Å, °)

*D*—H⋯*A*	*D*—H	H⋯*A*	*D*⋯*A*	*D*—H⋯*A*
C3—H3⋯O1^i^	0.93	2.56	3.460 (10)	164
C18—H18*C*⋯O5^ii^	0.96	2.55	3.421 (14)	151
C24—H24⋯O3	0.93	2.57	3.421 (13)	153
C42—H42*C*⋯O6^iii^	0.96	2.58	3.336 (16)	136

Symmetry codes: (i) 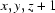; (ii) 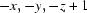; (iii) 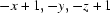.

**Fig. 1.**
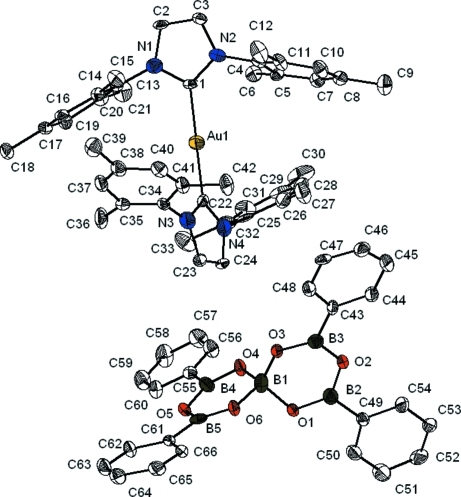
A molecular structure of the title compound with the atom-numbering scheme. Displacement ellipsoids are drawn at the 30 % probability level.

## supplementary crystallographic information

### Comment

The title compound was obtained as a side product in the synthesis of the (1,3-dimesitylimidazolium)gold phenyl complex in an attempt to transmetallate the NHC gold(I) *tert*-butoxide (Johnson *et al.*, 2011) using phenyl boronic acid. A different approach leading to the NHC gold(I) phenyl complex was reported by Pazicky *et al.* (2010).

The gold atom in compound (I) adopts a slightly distorted linear coordination geometry with a C1—Au—C22 angle of 176.8 (3)°. The coordination of two neutral ligands results in a delocalized positive charge which is balanced by the spiro tetraphenylpentaborate anion. The Au—C1 and Au—C22 coordination bonds of 1.982 (9) Å and 1.989 (9) Å, respectively, do not differ significantly with each other, but are slightly shorter than for example its 1,3-dimethylbenzimidazolium analogue at 2.054 (9) Å (Wang *et al.* 2005). This is as expected for a less electron deficient ligand. In the tetraphenylpentaborate anion, the boroxine rings are both flat due to a partial delocalization of electrons which is a result of π–π interactions between the filled *p*-orbitals of oxygen and vacant *p*-orbitals of boron (Haddon *et al.* 1982). Regarding the tetracoordinate boron, the four B—O bonds are in the range 1.43 (1)–1.47 (1) Å, in excellent agreement with the *m*-xylyl analogue 1.45 (2)–1.49 (1) Å (Nishihara *et al.* 2004). There is a weak C–H···O intermolecular interaction [H···O = 2.47–2.60 Å; C–H···O = 132–155°].

### Experimental

Under inert conditions, IMesAuOtBu (100 mg, 0.18 mmol) was dissolved in 5 ml toluene. PhB(OH)_2_ (42 mg, 0.35 mmol) was added together with Cs_2_CO_3_ (114 mg, 0.35 mmol). The suspension was stirred in the dark at 50 °C for 24 h. After cooling to room temperature, the mixture was filtered through Celite and the volatiles were removed *in vacuo*. Recrystallization of the resulting powder left colourless plates of the title compound.

### Crystal data

**Table:**

[Au(C_21_H_24_N_2_)_2_](C_24_H_20_B_5_O_6_)	*Z* = 2
*M**_r_* = 1264.26	*F*(000) = 1288
Triclinic, *P*1	*D*_x_ = 1.362 Mg m^−^^3^
Hall symbol: -P 1	Mo *K*α radiation, λ = 0.71073 Å
*a* = 15.388 (2) Å	Cell parameters from 7585 reflections
*b* = 15.768 (2) Å	θ = 2.3–33.2°
*c* = 16.453 (2) Å	µ = 2.44 mm^−^^1^
α = 94.81 (1)°	*T* = 293 K
β = 112.86 (1)°	Plate, colorless
γ = 117.65 (1)°	0.20 × 0.20 × 0.10 mm
*V* = 3083.2 Å^3^	

### Data collection

**Table:**

Oxford Diffraction Xcalibur-3 CCD diffractometer	10639 independent reflections
Radiation source: Enhance (Mo) X-ray Source	7217 reflections with *I* > 2σ(*I*)
graphite	*R*_int_ = 0.051
Detector resolution: 16.1829 pixels mm^-1^	θ_max_ = 25.0°, θ_min_ = 2.3°
ω scans	*h* = −18→17
Absorption correction: multi-scan (*CrysAlis RED*; Oxford Diffraction, 2006)	*k* = −13→18
*T*_min_ = 0.838, *T*_max_ = 1.000	*l* = −19→19
19866 measured reflections	

### Refinement

**Table:**

Refinement on *F*^2^	Primary atom site location: structure-invariant direct methods
Least-squares matrix: full	Secondary atom site location: difference Fourier map
*R*[*F*^2^ > 2σ(*F*^2^)] = 0.046	Hydrogen site location: inferred from neighbouring sites
*wR*(*F*^2^) = 0.152	H-atom parameters constrained
*S* = 1.08	*w* = 1/[σ^2^(*F*_o_^2^) + (0.0797*P*)^2^] where *P* = (*F*_o_^2^ + 2*F*_c_^2^)/3
10639 reflections	(Δ/σ)_max_ = 0.002
751 parameters	Δρ_max_ = 1.02 e Å^−^^3^
0 restraints	Δρ_min_ = −0.91 e Å^−^^3^

### Special details

**Table:**

Geometry. All s.u.'s (except the s.u. in the dihedral angle between two l.s. planes) are estimated using the full covariance matrix. The cell s.u.'s are taken into account individually in the estimation of s.u.'s in distances, angles and torsion angles; correlations between s.u.'s in cell parameters are only used when they are defined by crystal symmetry. An approximate (isotropic) treatment of cell s.u.'s is used for estimating s.u.'s involving l.s. planes.
Refinement. Refinement of *F*^2^ against ALL reflections. The weighted *R*-factor *wR* and goodness of fit *S* are based on *F*^2^, conventional *R*-factors *R* are based on *F*, with *F* set to zero for negative *F*^2^. The threshold expression of *F*^2^ > 2σ(*F*^2^) is used only for calculating *R*-factors(gt) *etc*. and is not relevant to the choice of reflections for refinement. *R*-factors based on *F*^2^ are statistically about twice as large as those based on *F*, and *R*- factors based on ALL data will be even larger.

### Fractional atomic coordinates and isotropic or equivalent isotropic displacement parameters (Å^2^)

**Table:**

	*x*	*y*	*z*	*U*_iso_*/*U*_eq_	
Au1	0.38230 (3)	0.16116 (2)	0.80812 (2)	0.05262 (14)	
O5	0.2525 (4)	0.1693 (4)	0.2299 (3)	0.0546 (13)	
O6	0.3904 (4)	0.1308 (4)	0.2674 (4)	0.0603 (14)	
N2	0.4720 (5)	0.2319 (4)	1.0153 (4)	0.0499 (15)	
O2	0.7074 (4)	0.3338 (4)	0.4622 (4)	0.0607 (14)	
O3	0.5298 (4)	0.2002 (4)	0.4263 (4)	0.0581 (13)	
O4	0.4233 (4)	0.2732 (4)	0.3694 (4)	0.0710 (17)	
C1	0.3848 (6)	0.1842 (5)	0.9295 (5)	0.0443 (17)	
C49	0.7431 (7)	0.4448 (6)	0.3638 (6)	0.057 (2)	
O1	0.5531 (4)	0.2929 (4)	0.3195 (3)	0.0579 (13)	
N1	0.2940 (6)	0.1477 (5)	0.9393 (4)	0.0557 (16)	
C2	0.3269 (8)	0.1689 (6)	1.0337 (5)	0.060 (2)	
H2	0.2795	0.1488	1.0598	0.072*	
N3	0.3280 (6)	0.0399 (6)	0.6263 (5)	0.066 (2)	
C22	0.3783 (6)	0.1303 (6)	0.6865 (5)	0.0530 (19)	
C13	0.1819 (6)	0.0923 (6)	0.8632 (5)	0.0495 (18)	
C4	0.5847 (7)	0.2853 (6)	1.0355 (5)	0.0508 (19)	
N4	0.4279 (6)	0.1985 (6)	0.6507 (5)	0.070 (2)	
C24	0.4102 (7)	0.1509 (8)	0.5677 (6)	0.065 (2)	
H24	0.4375	0.1820	0.5300	0.079*	
C61	0.1974 (6)	−0.0002 (5)	0.1391 (5)	0.0483 (17)	
C14	0.1272 (7)	−0.0091 (6)	0.8245 (6)	0.060 (2)	
C7	0.7416 (8)	0.4358 (7)	1.0543 (7)	0.076 (3)	
H7	0.7770	0.5040	1.0593	0.091*	
B1	0.4750 (8)	0.2237 (7)	0.3479 (7)	0.062 (3)	
C34	0.2674 (7)	−0.0509 (7)	0.6360 (5)	0.057 (2)	
C3	0.4360 (8)	0.2220 (6)	1.0793 (6)	0.061 (2)	
H3	0.4809	0.2484	1.1437	0.073*	
B4	0.3235 (7)	0.2549 (7)	0.3065 (6)	0.053 (2)	
C55	0.2825 (7)	0.3239 (6)	0.3249 (6)	0.058 (2)	
C5	0.6320 (7)	0.3859 (6)	1.0311 (6)	0.055 (2)	
C43	0.6982 (6)	0.2235 (6)	0.5615 (6)	0.060 (2)	
C66	0.2276 (7)	−0.0671 (6)	0.1204 (5)	0.057 (2)	
H66	0.3027	−0.0461	0.1522	0.069*	
C16	0.0202 (8)	−0.0594 (7)	0.7499 (6)	0.072 (3)	
H16	−0.0189	−0.1293	0.7239	0.086*	
C47	0.6888 (9)	0.1052 (7)	0.6477 (6)	0.067 (2)	
H47	0.6463	0.0450	0.6561	0.081*	
B5	0.2866 (8)	0.1053 (6)	0.2154 (6)	0.046 (2)	
B2	0.6618 (8)	0.3522 (7)	0.3812 (7)	0.056 (2)	
C35	0.1545 (8)	−0.0956 (8)	0.6019 (6)	0.068 (2)	
C48	0.6397 (8)	0.1359 (7)	0.5772 (6)	0.071 (3)	
H48	0.5626	0.0949	0.5383	0.085*	
B3	0.6399 (8)	0.2525 (6)	0.4792 (6)	0.049 (2)	
C25	0.4923 (9)	0.3063 (6)	0.6965 (6)	0.066 (2)	
C20	0.1354 (8)	0.1461 (7)	0.8298 (6)	0.067 (2)	
C62	0.0859 (7)	−0.0347 (7)	0.0891 (6)	0.072 (2)	
H62	0.0621	0.0077	0.0993	0.086*	
C19	0.0285 (8)	0.0937 (9)	0.7512 (7)	0.081 (3)	
H19	−0.0029	0.1291	0.7245	0.097*	
C10	0.7521 (9)	0.2952 (8)	1.0674 (7)	0.087 (3)	
H10	0.7925	0.2644	1.0788	0.104*	
C60	0.1821 (8)	0.3076 (7)	0.2628 (7)	0.077 (3)	
H60	0.1347	0.2516	0.2090	0.092*	
C38	0.1547 (9)	−0.2332 (8)	0.6637 (7)	0.083 (3)	
C64	0.0418 (10)	−0.1930 (7)	0.0096 (7)	0.088 (3)	
H64	−0.0105	−0.2577	−0.0335	0.106*	
C17	−0.0299 (8)	−0.0094 (8)	0.7135 (6)	0.076 (3)	
C11	0.6429 (8)	0.2401 (7)	1.0479 (6)	0.073 (3)	
C26	0.6032 (10)	0.3520 (8)	0.7661 (8)	0.100 (4)	
C56	0.3499 (8)	0.4098 (7)	0.3988 (7)	0.087 (3)	
H56	0.4212	0.4255	0.4399	0.104*	
C40	0.2671 (9)	−0.1834 (8)	0.6961 (7)	0.080 (3)	
H40	0.3049	−0.2128	0.7261	0.096*	
C46	0.7995 (11)	0.1648 (10)	0.7031 (8)	0.094 (3)	
H46	0.8330	0.1424	0.7487	0.113*	
C50	0.7072 (9)	0.4704 (8)	0.2884 (7)	0.087 (3)	
H50	0.6320	0.4294	0.2435	0.104*	
C8	0.8040 (8)	0.3937 (8)	1.0708 (7)	0.077 (3)	
C53	0.9260 (9)	0.5923 (8)	0.4124 (8)	0.091 (3)	
H53	1.0022	0.6327	0.4551	0.110*	
C54	0.8563 (8)	0.5067 (7)	0.4283 (8)	0.087 (3)	
H54	0.8858	0.4916	0.4819	0.105*	
C41	0.3257 (7)	−0.0942 (7)	0.6868 (6)	0.074 (3)	
C15	0.1828 (9)	−0.0663 (7)	0.8671 (7)	0.087 (3)	
H15A	0.2378	−0.0563	0.8484	0.130*	
H15B	0.1266	−0.1372	0.8455	0.130*	
H15C	0.2185	−0.0410	0.9339	0.130*	
C45	0.8633 (10)	0.2509 (12)	0.6984 (8)	0.111 (4)	
H45	0.9394	0.2912	0.7411	0.134*	
C37	0.1021 (8)	−0.1886 (9)	0.6225 (7)	0.095 (4)	
H37	0.0267	−0.2191	0.6059	0.114*	
C65	0.1522 (10)	−0.1616 (7)	0.0577 (7)	0.083 (3)	
H65	0.1753	−0.2046	0.0476	0.099*	
C44	0.8110 (8)	0.2829 (8)	0.6232 (7)	0.080 (3)	
H44	0.8549	0.3443	0.6171	0.096*	
C63	0.0079 (8)	−0.1300 (7)	0.0244 (7)	0.088 (3)	
H63	−0.0671	−0.1511	−0.0089	0.105*	
C42	0.4486 (8)	−0.0419 (8)	0.7250 (8)	0.103 (4)	
H42A	0.4857	0.0169	0.7774	0.155*	
H42B	0.4677	−0.0218	0.6778	0.155*	
H42C	0.4720	−0.0868	0.7445	0.155*	
C32	0.4382 (11)	0.3563 (8)	0.6753 (7)	0.088 (3)	
C18	−0.1442 (8)	−0.0652 (10)	0.6316 (7)	0.113 (4)	
H18A	−0.1455	−0.1023	0.5814	0.170*	
H18B	−0.1640	−0.0181	0.6124	0.170*	
H18C	−0.1970	−0.1115	0.6482	0.170*	
C6	0.5682 (8)	0.4327 (7)	1.0191 (7)	0.079 (3)	
H6A	0.6088	0.4923	1.0718	0.119*	
H6B	0.4971	0.3860	1.0143	0.119*	
H6C	0.5565	0.4510	0.9634	0.119*	
C28	0.6574 (10)	0.4549 (9)	0.8149 (9)	0.122 (5)	
H28	0.7316	0.4901	0.8620	0.146*	
C57	0.3193 (11)	0.4744 (9)	0.4165 (9)	0.108 (4)	
H57	0.3658	0.5290	0.4714	0.129*	
C39	0.0928 (10)	−0.3329 (8)	0.6825 (9)	0.110 (4)	
H39A	0.0663	−0.3221	0.7239	0.165*	
H39B	0.1435	−0.3542	0.7105	0.165*	
H39C	0.0304	−0.3843	0.6247	0.165*	
C9	0.9243 (9)	0.4502 (11)	1.0940 (10)	0.127 (5)	
H9A	0.9701	0.4943	1.1574	0.190*	
H9B	0.9344	0.4894	1.0531	0.190*	
H9C	0.9457	0.4030	1.0863	0.190*	
C33	0.3176 (12)	0.3032 (11)	0.6007 (9)	0.138 (5)	
H33A	0.3132	0.3318	0.5511	0.207*	
H33B	0.2748	0.3114	0.6266	0.207*	
H33C	0.2880	0.2325	0.5767	0.207*	
C58	0.2200 (11)	0.4581 (10)	0.3530 (11)	0.121 (5)	
H58	0.2004	0.5047	0.3606	0.145*	
C21	0.1984 (10)	0.2590 (8)	0.8708 (9)	0.111 (4)	
H21A	0.2570	0.2806	0.9333	0.167*	
H21B	0.1480	0.2782	0.8716	0.167*	
H21C	0.2306	0.2904	0.8336	0.167*	
C59	0.1498 (10)	0.3724 (9)	0.2782 (9)	0.102 (4)	
H59	0.0789	0.3575	0.2369	0.122*	
C12	0.5882 (9)	0.1295 (7)	1.0455 (8)	0.108 (4)	
H12A	0.5485	0.1187	1.0803	0.162*	
H12B	0.6451	0.1148	1.0726	0.162*	
H12C	0.5367	0.0858	0.9821	0.162*	
C51	0.7768 (11)	0.5559 (9)	0.2732 (9)	0.120 (4)	
H51	0.7487	0.5710	0.2192	0.144*	
C27	0.6588 (10)	0.2926 (10)	0.7902 (9)	0.119 (4)	
H27A	0.7196	0.3178	0.7763	0.179*	
H27B	0.6049	0.2223	0.7542	0.179*	
H27C	0.6871	0.3001	0.8554	0.179*	
C31	0.4970 (14)	0.4580 (11)	0.7261 (10)	0.116 (4)	
H31	0.4623	0.4940	0.7123	0.140*	
C52	0.8854 (11)	0.6160 (8)	0.3386 (9)	0.109 (4)	
H52	0.9321	0.6750	0.3311	0.131*	
C29	0.6011 (16)	0.5039 (10)	0.7935 (10)	0.108 (4)	
C30	0.6642 (14)	0.6165 (8)	0.8543 (11)	0.165 (8)	
H30A	0.7294	0.6569	0.8481	0.247*	
H30B	0.6865	0.6206	0.9185	0.247*	
H30C	0.6153	0.6409	0.8338	0.247*	
C36	0.0911 (9)	−0.0539 (10)	0.5597 (9)	0.114 (4)	
H36A	0.1198	0.0093	0.6029	0.172*	
H36B	0.0140	−0.0999	0.5427	0.172*	
H36C	0.0965	−0.0427	0.5049	0.172*	
C23	0.3475 (7)	0.0527 (8)	0.5503 (6)	0.074 (3)	
H23	0.3213	0.0017	0.4979	0.089*	

### Atomic displacement parameters (Å^2^)

**Table:**

	*U*^11^	*U*^22^	*U*^33^	*U*^12^	*U*^13^	*U*^23^
Au1	0.0546 (2)	0.0573 (2)	0.04296 (19)	0.02929 (16)	0.02380 (14)	0.00809 (13)
O5	0.050 (3)	0.054 (3)	0.047 (3)	0.030 (3)	0.012 (2)	0.013 (2)
O6	0.040 (3)	0.059 (3)	0.066 (4)	0.026 (3)	0.015 (3)	0.001 (3)
N2	0.061 (4)	0.053 (4)	0.034 (3)	0.032 (3)	0.020 (3)	0.013 (3)
O2	0.043 (3)	0.061 (3)	0.056 (3)	0.021 (3)	0.016 (3)	0.012 (3)
O3	0.046 (3)	0.063 (3)	0.058 (3)	0.026 (3)	0.022 (3)	0.025 (3)
O4	0.045 (3)	0.066 (4)	0.073 (4)	0.026 (3)	0.014 (3)	−0.012 (3)
C1	0.059 (5)	0.044 (4)	0.035 (4)	0.028 (4)	0.026 (4)	0.018 (3)
C49	0.052 (5)	0.048 (4)	0.054 (5)	0.019 (4)	0.020 (4)	0.009 (4)
O1	0.044 (3)	0.063 (3)	0.047 (3)	0.021 (3)	0.015 (3)	0.017 (3)
N1	0.062 (4)	0.051 (4)	0.051 (4)	0.028 (3)	0.029 (4)	0.012 (3)
C2	0.081 (6)	0.067 (5)	0.043 (5)	0.035 (5)	0.041 (5)	0.034 (4)
N3	0.054 (4)	0.071 (5)	0.056 (4)	0.030 (4)	0.022 (3)	−0.009 (4)
C22	0.044 (4)	0.059 (5)	0.055 (5)	0.024 (4)	0.029 (4)	0.013 (4)
C13	0.057 (5)	0.058 (5)	0.038 (4)	0.030 (4)	0.026 (4)	0.022 (4)
C4	0.062 (5)	0.058 (5)	0.032 (4)	0.038 (4)	0.015 (4)	0.013 (3)
N4	0.052 (4)	0.080 (5)	0.063 (5)	0.033 (4)	0.025 (4)	−0.007 (4)
C24	0.071 (6)	0.092 (7)	0.048 (5)	0.044 (6)	0.041 (5)	0.026 (5)
C61	0.047 (4)	0.053 (4)	0.038 (4)	0.022 (4)	0.020 (3)	0.015 (3)
C14	0.065 (5)	0.054 (5)	0.058 (5)	0.029 (4)	0.030 (4)	0.018 (4)
C7	0.077 (7)	0.065 (6)	0.078 (7)	0.034 (6)	0.037 (5)	0.019 (5)
B1	0.042 (5)	0.056 (6)	0.075 (7)	0.021 (5)	0.025 (5)	0.008 (5)
C34	0.052 (5)	0.072 (6)	0.039 (4)	0.034 (5)	0.017 (4)	0.003 (4)
C3	0.069 (6)	0.062 (5)	0.041 (5)	0.032 (5)	0.022 (4)	0.017 (4)
B4	0.048 (5)	0.060 (6)	0.042 (5)	0.029 (5)	0.014 (4)	0.013 (4)
C55	0.066 (6)	0.053 (5)	0.058 (5)	0.032 (4)	0.032 (4)	0.022 (4)
C5	0.065 (6)	0.053 (5)	0.057 (5)	0.033 (4)	0.036 (4)	0.033 (4)
C43	0.040 (5)	0.058 (5)	0.067 (6)	0.020 (4)	0.025 (4)	0.008 (4)
C66	0.051 (5)	0.061 (5)	0.047 (5)	0.025 (4)	0.020 (4)	0.015 (4)
C16	0.073 (6)	0.055 (5)	0.048 (5)	0.002 (5)	0.036 (5)	0.022 (4)
C47	0.098 (8)	0.093 (7)	0.057 (5)	0.066 (6)	0.054 (6)	0.052 (5)
B5	0.069 (6)	0.049 (5)	0.038 (4)	0.033 (5)	0.037 (4)	0.031 (4)
B2	0.048 (6)	0.056 (6)	0.052 (6)	0.023 (5)	0.021 (5)	0.005 (4)
C35	0.068 (6)	0.087 (7)	0.052 (5)	0.041 (6)	0.031 (5)	0.030 (5)
C48	0.058 (5)	0.075 (6)	0.070 (6)	0.037 (5)	0.026 (5)	−0.004 (5)
B3	0.055 (6)	0.048 (5)	0.047 (5)	0.027 (5)	0.027 (5)	0.013 (4)
C25	0.103 (7)	0.062 (5)	0.060 (6)	0.037 (6)	0.068 (6)	0.040 (5)
C20	0.077 (6)	0.068 (6)	0.072 (6)	0.046 (5)	0.042 (5)	0.029 (5)
C62	0.051 (5)	0.063 (6)	0.071 (6)	0.025 (5)	0.015 (4)	0.004 (5)
C19	0.065 (6)	0.101 (8)	0.081 (7)	0.052 (6)	0.031 (6)	0.031 (6)
C10	0.072 (7)	0.092 (8)	0.088 (7)	0.057 (6)	0.020 (6)	0.014 (6)
C60	0.061 (6)	0.061 (5)	0.097 (7)	0.033 (5)	0.031 (5)	0.014 (5)
C38	0.076 (7)	0.078 (7)	0.069 (6)	0.036 (6)	0.025 (5)	−0.004 (5)
C64	0.085 (8)	0.056 (6)	0.073 (7)	0.013 (6)	0.029 (6)	−0.006 (5)
C17	0.067 (6)	0.103 (8)	0.055 (6)	0.040 (6)	0.029 (5)	0.046 (6)
C11	0.064 (6)	0.065 (6)	0.068 (6)	0.041 (5)	0.008 (5)	0.001 (4)
C26	0.079 (8)	0.087 (8)	0.091 (8)	0.016 (6)	0.049 (7)	−0.008 (6)
C56	0.077 (7)	0.077 (6)	0.086 (7)	0.044 (6)	0.023 (5)	0.000 (5)
C40	0.089 (8)	0.073 (7)	0.072 (6)	0.051 (6)	0.029 (6)	0.005 (5)
C46	0.091 (9)	0.127 (10)	0.094 (9)	0.068 (8)	0.054 (8)	0.059 (8)
C50	0.075 (7)	0.077 (7)	0.067 (7)	0.023 (6)	0.021 (5)	0.022 (5)
C8	0.073 (6)	0.097 (8)	0.066 (6)	0.048 (6)	0.033 (5)	0.031 (5)
C53	0.073 (7)	0.081 (7)	0.072 (7)	0.007 (6)	0.037 (6)	0.026 (6)
C54	0.065 (6)	0.076 (7)	0.081 (7)	0.014 (5)	0.034 (6)	0.007 (5)
C41	0.059 (6)	0.068 (6)	0.074 (6)	0.033 (5)	0.022 (5)	−0.012 (5)
C15	0.096 (8)	0.068 (6)	0.080 (7)	0.045 (6)	0.028 (6)	0.026 (5)
C45	0.063 (7)	0.159 (12)	0.092 (9)	0.058 (8)	0.021 (6)	0.049 (9)
C37	0.053 (6)	0.129 (10)	0.070 (7)	0.045 (7)	0.016 (5)	−0.010 (7)
C65	0.099 (8)	0.072 (6)	0.067 (6)	0.042 (6)	0.040 (6)	0.003 (5)
C44	0.060 (6)	0.093 (7)	0.078 (7)	0.034 (5)	0.031 (5)	0.048 (6)
C63	0.061 (6)	0.071 (6)	0.079 (7)	0.019 (5)	0.016 (5)	−0.003 (5)
C42	0.067 (7)	0.103 (8)	0.129 (10)	0.060 (6)	0.027 (6)	0.010 (7)
C32	0.132 (10)	0.081 (7)	0.073 (7)	0.062 (7)	0.058 (7)	0.040 (6)
C18	0.059 (6)	0.164 (12)	0.083 (8)	0.043 (7)	0.021 (6)	0.066 (8)
C6	0.090 (7)	0.068 (6)	0.088 (7)	0.047 (6)	0.043 (6)	0.038 (5)
C28	0.082 (8)	0.094 (9)	0.118 (10)	−0.001 (7)	0.061 (7)	−0.034 (8)
C57	0.119 (10)	0.101 (8)	0.101 (9)	0.071 (8)	0.041 (8)	0.005 (7)
C39	0.098 (9)	0.077 (7)	0.120 (10)	0.030 (7)	0.047 (7)	0.014 (7)
C9	0.076 (8)	0.149 (12)	0.136 (12)	0.052 (8)	0.046 (8)	0.043 (9)
C33	0.144 (13)	0.153 (12)	0.114 (11)	0.109 (11)	0.027 (9)	0.030 (9)
C58	0.103 (10)	0.102 (9)	0.161 (13)	0.071 (8)	0.053 (9)	0.009 (9)
C21	0.123 (10)	0.070 (7)	0.144 (11)	0.059 (7)	0.058 (8)	0.037 (7)
C59	0.085 (8)	0.114 (9)	0.119 (10)	0.073 (8)	0.038 (7)	0.027 (8)
C12	0.101 (8)	0.067 (6)	0.127 (10)	0.058 (6)	0.019 (7)	0.014 (6)
C51	0.105 (9)	0.091 (8)	0.098 (9)	0.015 (8)	0.034 (8)	0.053 (7)
C27	0.083 (8)	0.124 (10)	0.114 (10)	0.060 (8)	0.020 (7)	−0.001 (8)
C31	0.150 (13)	0.117 (11)	0.110 (11)	0.090 (11)	0.064 (10)	0.040 (9)
C52	0.118 (10)	0.073 (7)	0.098 (9)	0.011 (7)	0.068 (8)	0.036 (7)
C29	0.177 (14)	0.098 (9)	0.104 (10)	0.071 (10)	0.114 (11)	0.055 (8)
C30	0.26 (2)	0.066 (8)	0.164 (14)	0.043 (10)	0.158 (15)	0.020 (8)
C36	0.056 (7)	0.138 (10)	0.133 (11)	0.053 (7)	0.034 (7)	0.026 (8)
C23	0.061 (6)	0.104 (8)	0.053 (5)	0.041 (6)	0.032 (4)	−0.003 (5)

### Geometric parameters (Å, °)

**Table:**

Au1—C1	1.982 (7)	C38—C39	1.547 (15)
Au1—C22	1.990 (8)	C64—C63	1.363 (13)
O5—B4	1.370 (10)	C64—C65	1.366 (14)
O5—B5	1.378 (9)	C64—H64	0.9300
O6—B5	1.315 (10)	C17—C18	1.486 (13)
O6—B1	1.470 (10)	C11—C12	1.533 (13)
N2—C1	1.331 (9)	C26—C28	1.407 (15)
N2—C3	1.356 (10)	C26—C27	1.519 (16)
N2—C4	1.407 (10)	C56—C57	1.358 (13)
O2—B3	1.360 (10)	C56—H56	0.9300
O2—B2	1.371 (11)	C40—C41	1.334 (14)
O3—B3	1.321 (10)	C40—H40	0.9300
O3—B1	1.427 (12)	C46—C45	1.285 (16)
O4—B4	1.348 (10)	C46—H46	0.9300
O4—B1	1.453 (10)	C50—C51	1.397 (14)
C1—N1	1.324 (9)	C50—H50	0.9300
C49—C50	1.329 (12)	C8—C9	1.493 (14)
C49—C54	1.387 (12)	C53—C52	1.307 (15)
C49—B2	1.560 (13)	C53—C54	1.406 (14)
O1—B2	1.331 (10)	C53—H53	0.9300
O1—B1	1.470 (12)	C54—H54	0.9300
N1—C2	1.388 (9)	C41—C42	1.480 (12)
N1—C13	1.430 (10)	C15—H15A	0.9600
C2—C3	1.302 (11)	C15—H15B	0.9600
C2—H2	0.9300	C15—H15C	0.9600
N3—C22	1.324 (9)	C45—C44	1.463 (14)
N3—C34	1.365 (11)	C45—H45	0.9300
N3—C23	1.404 (11)	C37—H37	0.9300
C22—N4	1.334 (11)	C65—H65	0.9300
C13—C20	1.368 (11)	C44—H44	0.9300
C13—C14	1.354 (10)	C63—H63	0.9300
C4—C11	1.352 (10)	C42—H42A	0.9600
C4—C5	1.428 (11)	C42—H42B	0.9600
N4—C24	1.361 (10)	C42—H42C	0.9600
N4—C25	1.443 (11)	C32—C31	1.390 (16)
C24—C23	1.317 (12)	C32—C33	1.506 (16)
C24—H24	0.9300	C18—H18A	0.9600
C61—C62	1.372 (11)	C18—H18B	0.9600
C61—C66	1.390 (10)	C18—H18C	0.9600
C61—B5	1.555 (11)	C6—H6A	0.9600
C14—C16	1.379 (12)	C6—H6B	0.9600
C14—C15	1.547 (12)	C6—H6C	0.9600
C7—C5	1.352 (12)	C28—C29	1.378 (18)
C7—C8	1.363 (13)	C28—H28	0.9300
C7—H7	0.9300	C57—C58	1.358 (16)
C34—C35	1.366 (11)	C57—H57	0.9300
C34—C41	1.432 (12)	C39—H39A	0.9600
C3—H3	0.9300	C39—H39B	0.9600
B4—C55	1.551 (11)	C39—H39C	0.9600
C55—C56	1.352 (11)	C9—H9A	0.9600
C55—C60	1.364 (12)	C9—H9B	0.9600
C5—C6	1.446 (11)	C9—H9C	0.9600
C43—C44	1.372 (11)	C33—H33A	0.9600
C43—C48	1.367 (12)	C33—H33B	0.9600
C43—B3	1.546 (12)	C33—H33C	0.9600
C66—C65	1.356 (11)	C58—C59	1.356 (15)
C66—H66	0.9300	C58—H58	0.9300
C16—C17	1.363 (13)	C21—H21A	0.9600
C16—H16	0.9300	C21—H21B	0.9600
C47—C46	1.334 (14)	C21—H21C	0.9600
C47—C48	1.380 (12)	C59—H59	0.9300
C47—H47	0.9300	C12—H12A	0.9600
C35—C36	1.420 (14)	C12—H12B	0.9600
C35—C37	1.453 (15)	C12—H12C	0.9600
C48—H48	0.9300	C51—C52	1.345 (16)
C25—C32	1.365 (13)	C51—H51	0.9300
C25—C26	1.395 (14)	C27—H27A	0.9600
C20—C19	1.403 (13)	C27—H27B	0.9600
C20—C21	1.501 (13)	C27—H27C	0.9600
C62—C63	1.378 (12)	C31—C29	1.315 (18)
C62—H62	0.9300	C31—H31	0.9300
C19—C17	1.370 (13)	C52—H52	0.9300
C19—H19	0.9300	C29—C30	1.563 (16)
C10—C11	1.363 (13)	C30—H30A	0.9600
C10—C8	1.361 (14)	C30—H30B	0.9600
C10—H10	0.9300	C30—H30C	0.9600
C60—C59	1.372 (12)	C36—H36A	0.9600
C60—H60	0.9300	C36—H36B	0.9600
C38—C37	1.338 (14)	C36—H36C	0.9600
C38—C40	1.359 (13)	C23—H23	0.9300
			
C1—Au1—C22	176.8 (3)	C45—C46—C47	124.9 (11)
B4—O5—B5	119.4 (6)	C45—C46—H46	117.5
B5—O6—B1	123.6 (6)	C47—C46—H46	117.5
C1—N2—C3	110.2 (7)	C49—C50—C51	123.3 (10)
C1—N2—C4	124.3 (6)	C49—C50—H50	118.4
C3—N2—C4	125.5 (6)	C51—C50—H50	118.4
B3—O2—B2	119.1 (7)	C10—C8—C7	116.7 (9)
B3—O3—B1	122.7 (7)	C10—C8—C9	119.7 (10)
B4—O4—B1	122.3 (7)	C7—C8—C9	123.6 (11)
N2—C1—N1	106.3 (6)	C52—C53—C54	120.9 (11)
N2—C1—Au1	129.3 (6)	C52—C53—H53	119.5
N1—C1—Au1	124.3 (5)	C54—C53—H53	119.5
C50—C49—C54	116.7 (9)	C49—C54—C53	119.8 (11)
C50—C49—B2	122.0 (8)	C49—C54—H54	120.1
C54—C49—B2	121.3 (9)	C53—C54—H54	120.1
B2—O1—B1	119.5 (7)	C40—C41—C34	118.9 (9)
C1—N1—C2	108.5 (7)	C40—C41—C42	121.1 (10)
C1—N1—C13	124.2 (6)	C34—C41—C42	120.0 (10)
C2—N1—C13	127.2 (7)	C14—C15—H15A	109.5
C3—C2—N1	107.6 (7)	C14—C15—H15B	109.5
C3—C2—H2	126.2	H15A—C15—H15B	109.5
N1—C2—H2	126.2	C14—C15—H15C	109.5
C22—N3—C34	126.1 (7)	H15A—C15—H15C	109.5
C22—N3—C23	108.7 (8)	H15B—C15—H15C	109.5
C34—N3—C23	125.2 (7)	C46—C45—C44	117.2 (11)
N3—C22—N4	106.9 (7)	C46—C45—H45	121.4
N3—C22—Au1	127.4 (7)	C44—C45—H45	121.4
N4—C22—Au1	125.7 (6)	C38—C37—C35	124.5 (10)
C20—C13—C14	122.3 (8)	C38—C37—H37	117.7
C20—C13—N1	117.4 (7)	C35—C37—H37	117.7
C14—C13—N1	120.2 (7)	C64—C65—C66	119.2 (9)
C11—C4—N2	121.6 (8)	C64—C65—H65	120.4
C11—C4—C5	120.4 (8)	C66—C65—H65	120.4
N2—C4—C5	117.5 (6)	C43—C44—C45	120.8 (10)
C22—N4—C24	109.9 (7)	C43—C44—H44	119.6
C22—N4—C25	123.5 (7)	C45—C44—H44	119.6
C24—N4—C25	126.6 (8)	C64—C63—C62	119.3 (9)
C23—C24—N4	107.8 (8)	C64—C63—H63	120.3
C23—C24—H24	126.1	C62—C63—H63	120.3
N4—C24—H24	126.1	C41—C42—H42A	109.5
C62—C61—C66	116.1 (7)	C41—C42—H42B	109.5
C62—C61—B5	123.3 (7)	H42A—C42—H42B	109.5
C66—C61—B5	120.6 (7)	C41—C42—H42C	109.5
C16—C14—C13	118.4 (8)	H42A—C42—H42C	109.5
C16—C14—C15	121.6 (8)	H42B—C42—H42C	109.5
C13—C14—C15	119.9 (8)	C25—C32—C31	118.1 (12)
C5—C7—C8	124.8 (9)	C25—C32—C33	121.6 (10)
C5—C7—H7	117.6	C31—C32—C33	120.3 (12)
C8—C7—H7	117.6	C17—C18—H18A	109.5
O3—B1—O4	110.9 (8)	C17—C18—H18B	109.5
O3—B1—O6	109.8 (7)	H18A—C18—H18B	109.5
O4—B1—O6	110.4 (6)	C17—C18—H18C	109.5
O3—B1—O1	112.2 (7)	H18A—C18—H18C	109.5
O4—B1—O1	106.7 (7)	H18B—C18—H18C	109.5
O6—B1—O1	106.7 (7)	C5—C6—H6A	109.5
C35—C34—N3	119.8 (8)	C5—C6—H6B	109.5
C35—C34—C41	121.7 (9)	H6A—C6—H6B	109.5
N3—C34—C41	118.5 (8)	C5—C6—H6C	109.5
C2—C3—N2	107.3 (7)	H6A—C6—H6C	109.5
C2—C3—H3	126.4	H6B—C6—H6C	109.5
N2—C3—H3	126.4	C29—C28—C26	120.4 (13)
O4—B4—O5	120.1 (7)	C29—C28—H28	119.8
O4—B4—C55	120.5 (7)	C26—C28—H28	119.8
O5—B4—C55	119.2 (7)	C58—C57—C56	119.1 (11)
C56—C55—C60	116.2 (8)	C58—C57—H57	120.4
C56—C55—B4	121.4 (8)	C56—C57—H57	120.4
C60—C55—B4	122.0 (7)	C38—C39—H39A	109.5
C7—C5—C6	124.4 (8)	C38—C39—H39B	109.5
C7—C5—C4	115.6 (7)	H39A—C39—H39B	109.5
C6—C5—C4	119.1 (8)	C38—C39—H39C	109.5
C44—C43—C48	115.7 (9)	H39A—C39—H39C	109.5
C44—C43—B3	122.7 (8)	H39B—C39—H39C	109.5
C48—C43—B3	121.6 (8)	C8—C9—H9A	109.5
C65—C66—C61	122.8 (8)	C8—C9—H9B	109.5
C65—C66—H66	118.6	H9A—C9—H9B	109.5
C61—C66—H66	118.6	C8—C9—H9C	109.5
C14—C16—C17	121.7 (9)	H9A—C9—H9C	109.5
C14—C16—H16	119.2	H9B—C9—H9C	109.5
C17—C16—H16	119.2	C32—C33—H33A	109.5
C46—C47—C48	117.6 (10)	C32—C33—H33B	109.5
C46—C47—H47	121.2	H33A—C33—H33B	109.5
C48—C47—H47	121.2	C32—C33—H33C	109.5
O6—B5—O5	120.9 (7)	H33A—C33—H33C	109.5
O6—B5—C61	120.8 (7)	H33B—C33—H33C	109.5
O5—B5—C61	118.2 (7)	C59—C58—C57	118.6 (10)
O1—B2—O2	121.5 (8)	C59—C58—H58	120.7
O1—B2—C49	121.4 (8)	C57—C58—H58	120.7
O2—B2—C49	117.1 (7)	C20—C21—H21A	109.5
C34—C35—C36	126.0 (10)	C20—C21—H21B	109.5
C34—C35—C37	113.9 (8)	H21A—C21—H21B	109.5
C36—C35—C37	119.7 (9)	C20—C21—H21C	109.5
C43—C48—C47	123.6 (9)	H21A—C21—H21C	109.5
C43—C48—H48	118.2	H21B—C21—H21C	109.5
C47—C48—H48	118.2	C58—C59—C60	120.8 (10)
O3—B3—O2	121.0 (8)	C58—C59—H59	119.6
O3—B3—C43	122.0 (8)	C60—C59—H59	119.6
O2—B3—C43	116.9 (7)	C11—C12—H12A	109.5
C32—C25—C26	123.6 (10)	C11—C12—H12B	109.5
C32—C25—N4	118.0 (10)	H12A—C12—H12B	109.5
C26—C25—N4	118.2 (9)	C11—C12—H12C	109.5
C13—C20—C19	118.1 (8)	H12A—C12—H12C	109.5
C13—C20—C21	121.9 (9)	H12B—C12—H12C	109.5
C19—C20—C21	119.9 (9)	C52—C51—C50	118.5 (11)
C61—C62—C63	122.2 (8)	C52—C51—H51	120.8
C61—C62—H62	118.9	C50—C51—H51	120.8
C63—C62—H62	118.9	C26—C27—H27A	109.5
C17—C19—C20	120.3 (9)	C26—C27—H27B	109.5
C17—C19—H19	119.8	H27A—C27—H27B	109.5
C20—C19—H19	119.8	C26—C27—H27C	109.5
C11—C10—C8	122.5 (9)	H27A—C27—H27C	109.5
C11—C10—H10	118.8	H27B—C27—H27C	109.5
C8—C10—H10	118.8	C29—C31—C32	120.6 (13)
C55—C60—C59	121.2 (9)	C29—C31—H31	119.7
C55—C60—H60	119.4	C32—C31—H31	119.7
C59—C60—H60	119.4	C53—C52—C51	120.8 (10)
C37—C38—C40	117.6 (11)	C53—C52—H52	119.6
C37—C38—C39	121.6 (11)	C51—C52—H52	119.6
C40—C38—C39	120.5 (11)	C31—C29—C28	122.0 (13)
C63—C64—C65	120.4 (9)	C31—C29—C30	120.3 (15)
C63—C64—H64	119.8	C28—C29—C30	117.7 (16)
C65—C64—H64	119.8	C29—C30—H30A	109.5
C19—C17—C16	119.0 (9)	C29—C30—H30B	109.5
C19—C17—C18	120.4 (10)	H30A—C30—H30B	109.5
C16—C17—C18	120.5 (10)	C29—C30—H30C	109.5
C4—C11—C10	119.3 (9)	H30A—C30—H30C	109.5
C4—C11—C12	119.9 (9)	H30B—C30—H30C	109.5
C10—C11—C12	120.6 (8)	C35—C36—H36A	109.5
C25—C26—C28	115.3 (12)	C35—C36—H36B	109.5
C25—C26—C27	121.9 (10)	H36A—C36—H36B	109.5
C28—C26—C27	122.7 (12)	C35—C36—H36C	109.5
C55—C56—C57	123.7 (10)	H36A—C36—H36C	109.5
C55—C56—H56	118.2	H36B—C36—H36C	109.5
C57—C56—H56	118.2	C24—C23—N3	106.6 (7)
C41—C40—C38	123.1 (10)	C24—C23—H23	126.7
C41—C40—H40	118.5	N3—C23—H23	126.7
C38—C40—H40	118.5		
			
C3—N2—C1—N1	2.4 (8)	C44—C43—C48—C47	−2.3 (12)
C4—N2—C1—N1	−177.0 (6)	B3—C43—C48—C47	177.6 (7)
C3—N2—C1—Au1	−174.1 (5)	C46—C47—C48—C43	0.1 (13)
C4—N2—C1—Au1	6.4 (10)	B1—O3—B3—O2	1.1 (11)
C22—Au1—C1—N2	96 (5)	B1—O3—B3—C43	−177.6 (7)
C22—Au1—C1—N1	−80 (6)	B2—O2—B3—O3	−9.8 (11)
N2—C1—N1—C2	−3.5 (8)	B2—O2—B3—C43	168.9 (7)
Au1—C1—N1—C2	173.3 (5)	C44—C43—B3—O3	−172.8 (8)
N2—C1—N1—C13	179.0 (6)	C48—C43—B3—O3	7.3 (11)
Au1—C1—N1—C13	−4.2 (10)	C44—C43—B3—O2	8.5 (11)
C1—N1—C2—C3	3.4 (9)	C48—C43—B3—O2	−171.4 (7)
C13—N1—C2—C3	−179.2 (7)	C22—N4—C25—C32	−92.8 (10)
C34—N3—C22—N4	180.0 (7)	C24—N4—C25—C32	88.7 (10)
C23—N3—C22—N4	0.2 (9)	C22—N4—C25—C26	81.4 (10)
C34—N3—C22—Au1	0.6 (12)	C24—N4—C25—C26	−97.1 (11)
C23—N3—C22—Au1	−179.1 (6)	C14—C13—C20—C19	−1.6 (13)
C1—Au1—C22—N3	24 (6)	N1—C13—C20—C19	175.7 (8)
C1—Au1—C22—N4	−155 (5)	C14—C13—C20—C21	−177.5 (9)
C1—N1—C13—C20	−90.9 (9)	N1—C13—C20—C21	−0.2 (13)
C2—N1—C13—C20	92.1 (10)	C66—C61—C62—C63	0.2 (14)
C1—N1—C13—C14	86.5 (10)	B5—C61—C62—C63	−177.5 (9)
C2—N1—C13—C14	−90.5 (9)	C13—C20—C19—C17	4.3 (14)
C1—N2—C4—C11	−91.3 (9)	C21—C20—C19—C17	−179.7 (10)
C3—N2—C4—C11	89.4 (10)	C56—C55—C60—C59	−3.7 (15)
C1—N2—C4—C5	81.2 (9)	B4—C55—C60—C59	−176.7 (10)
C3—N2—C4—C5	−98.1 (9)	C20—C19—C17—C16	−4.0 (15)
N3—C22—N4—C24	−0.9 (9)	C20—C19—C17—C18	178.5 (9)
Au1—C22—N4—C24	178.4 (5)	C14—C16—C17—C19	0.9 (14)
N3—C22—N4—C25	−179.6 (8)	C14—C16—C17—C18	178.4 (9)
Au1—C22—N4—C25	−0.2 (12)	N2—C4—C11—C10	−180.0 (8)
C22—N4—C24—C23	1.3 (10)	C5—C4—C11—C10	7.8 (13)
C25—N4—C24—C23	179.9 (9)	N2—C4—C11—C12	−3.4 (12)
C20—C13—C14—C16	−1.3 (12)	C5—C4—C11—C12	−175.7 (8)
N1—C13—C14—C16	−178.6 (7)	C8—C10—C11—C4	−3.1 (15)
C20—C13—C14—C15	−178.3 (9)	C8—C10—C11—C12	−179.6 (10)
N1—C13—C14—C15	4.4 (12)	C32—C25—C26—C28	0.5 (15)
B3—O3—B1—O4	−104.5 (8)	N4—C25—C26—C28	−173.3 (9)
B3—O3—B1—O6	133.2 (7)	C32—C25—C26—C27	177.4 (10)
B3—O3—B1—O1	14.7 (10)	N4—C25—C26—C27	3.6 (15)
B4—O4—B1—O3	−142.2 (8)	C60—C55—C56—C57	4.8 (16)
B4—O4—B1—O6	−20.3 (12)	B4—C55—C56—C57	177.9 (11)
B4—O4—B1—O1	95.3 (9)	C37—C38—C40—C41	−1.4 (14)
B5—O6—B1—O3	131.3 (7)	C39—C38—C40—C41	−175.9 (9)
B5—O6—B1—O4	8.7 (12)	C48—C47—C46—C45	3.1 (17)
B5—O6—B1—O1	−106.8 (8)	C54—C49—C50—C51	−1.3 (16)
B2—O1—B1—O3	−23.1 (10)	B2—C49—C50—C51	176.6 (10)
B2—O1—B1—O4	98.6 (8)	C11—C10—C8—C7	0.5 (15)
B2—O1—B1—O6	−143.4 (7)	C11—C10—C8—C9	179.1 (10)
C22—N3—C34—C35	90.5 (10)	C5—C7—C8—C10	−3.0 (15)
C23—N3—C34—C35	−89.7 (10)	C5—C7—C8—C9	178.4 (10)
C22—N3—C34—C41	−86.1 (10)	C50—C49—C54—C53	0.9 (14)
C23—N3—C34—C41	93.7 (10)	B2—C49—C54—C53	−177.0 (9)
N1—C2—C3—N2	−1.8 (9)	C52—C53—C54—C49	1.6 (17)
C1—N2—C3—C2	−0.3 (9)	C38—C40—C41—C34	−3.5 (14)
C4—N2—C3—C2	179.1 (7)	C38—C40—C41—C42	178.7 (9)
B1—O4—B4—O5	18.6 (13)	C35—C34—C41—C40	4.0 (12)
B1—O4—B4—C55	−167.8 (8)	N3—C34—C41—C40	−179.5 (7)
B5—O5—B4—O4	−3.4 (12)	C35—C34—C41—C42	−178.2 (8)
B5—O5—B4—C55	−177.1 (7)	N3—C34—C41—C42	−1.7 (11)
O4—B4—C55—C56	7.2 (14)	C47—C46—C45—C44	−3.5 (19)
O5—B4—C55—C56	−179.1 (9)	C40—C38—C37—C35	6.3 (15)
O4—B4—C55—C60	179.9 (9)	C39—C38—C37—C35	−179.3 (9)
O5—B4—C55—C60	−6.4 (13)	C34—C35—C37—C38	−5.6 (14)
C8—C7—C5—C6	176.1 (9)	C36—C35—C37—C38	−178.7 (10)
C8—C7—C5—C4	7.4 (14)	C63—C64—C65—C66	0.0 (16)
C11—C4—C5—C7	−9.7 (11)	C61—C66—C65—C64	0.8 (14)
N2—C4—C5—C7	177.7 (7)	C48—C43—C44—C45	1.8 (14)
C11—C4—C5—C6	−179.1 (8)	B3—C43—C44—C45	−178.1 (9)
N2—C4—C5—C6	8.3 (11)	C46—C45—C44—C43	1.0 (17)
C62—C61—C66—C65	−0.9 (12)	C65—C64—C63—C62	−0.7 (17)
B5—C61—C66—C65	176.9 (8)	C61—C62—C63—C64	0.6 (16)
C13—C14—C16—C17	1.7 (13)	C26—C25—C32—C31	−0.2 (15)
C15—C14—C16—C17	178.6 (9)	N4—C25—C32—C31	173.6 (9)
B1—O6—B5—O5	4.7 (12)	C26—C25—C32—C33	−178.9 (11)
B1—O6—B5—C61	−172.2 (8)	N4—C25—C32—C33	−5.1 (15)
B4—O5—B5—O6	−8.1 (11)	C25—C26—C28—C29	0.4 (17)
B4—O5—B5—C61	168.8 (7)	C27—C26—C28—C29	−176.5 (11)
C62—C61—B5—O6	173.4 (8)	C55—C56—C57—C58	−6(2)
C66—C61—B5—O6	−4.2 (11)	C56—C57—C58—C59	6(2)
C62—C61—B5—O5	−3.5 (11)	C57—C58—C59—C60	−5(2)
C66—C61—B5—O5	178.8 (7)	C55—C60—C59—C58	4.1 (19)
B1—O1—B2—O2	16.4 (11)	C49—C50—C51—C52	−1(2)
B1—O1—B2—C49	−164.3 (7)	C25—C32—C31—C29	−1.0 (19)
B3—O2—B2—O1	0.7 (11)	C33—C32—C31—C29	177.7 (13)
B3—O2—B2—C49	−178.6 (6)	C54—C53—C52—C51	−4(2)
C50—C49—B2—O1	2.0 (13)	C50—C51—C52—C53	3(2)
C54—C49—B2—O1	179.7 (8)	C32—C31—C29—C28	2(2)
C50—C49—B2—O2	−178.7 (8)	C32—C31—C29—C30	−176.2 (11)
C54—C49—B2—O2	−0.9 (12)	C26—C28—C29—C31	−2(2)
N3—C34—C35—C36	−3.6 (14)	C26—C28—C29—C30	176.6 (10)
C41—C34—C35—C36	172.9 (9)	N4—C24—C23—N3	−1.1 (10)
N3—C34—C35—C37	−176.2 (7)	C22—N3—C23—C24	0.6 (10)
C41—C34—C35—C37	0.3 (12)	C34—N3—C23—C24	−179.2 (8)

### Hydrogen-bond geometry (Å, °)

**Table:**

*D*—H···*A*	*D*—H	H···*A*	*D*···*A*	*D*—H···*A*
C3—H3···O1^i^	0.93	2.56	3.460 (10)	164
C18—H18C···O5^ii^	0.96	2.55	3.421 (14)	151
C24—H24···O3	0.93	2.57	3.421 (13)	153
C42—H42C···O6^iii^	0.96	2.58	3.336 (16)	136

Symmetry codes: (i) *x*, *y*, *z*+1; (ii) −*x*, −*y*, −*z*+1; (iii) −*x*+1, −*y*, −*z*+1.

## References

[bb1] Altomare, A., Burla, M. C., Camalli, M., Cascarano, G. L., Giacovazzo, C., Guagliardi, A., Moliterni, A. G. G., Polidori, G. & Spagna, R. (1999). *J. Appl. Cryst.* **32**, 115–119.

[bb2] Baker, M. V., Barnard, P. J., Berners-Price, S. J., Brayshaw, S. K., Hickey, J. L., Skelton, B. W. & White, A. H. (2006). *Dalton Trans.* pp. 3708–3715. 10.1039/b602560a 16865184

[bb3] Barnard, P. J. & Berners-Price, S. J. (2007). *Coord. Chem. Rev.* **251**, 1889–1902.

[bb4] Brandenburg, K. (2006). *DIAMOND* Crystal Impact GbR, Bonn, Germany.

[bb5] Gaillard, S., Nun, P., Slawin, A. M. Z. & Nolan, S. P. (2010). *Organometallics*, **29**, 5402–5408.

[bb6] Haddon, R. C. (1982). *Pure Appl. Chem.* **54**, 1129–1142.

[bb7] Hickey, J. L., Ruhayel, R. A., Barnard, P. J., Baker, M. V., Berners-Price, S. J. & Filipovska, A. (2008). *J. Am. Chem. Soc.* **130**, 12570–12571. 10.1021/ja804027j 18729360

[bb8] Johnson, M. T., van Rensburg, J. M. J., Axelsson, M., Ahlquist, M. S. G. & Wendt, O. F. (2011). In preparation.

[bb9] Nishihara, Y., Nara, K., Nishide, Y. & Osakada, K. (2004). *Dalton Trans.* pp. 1366–1375. 10.1039/b315902g 15252629

[bb10] Oxford Diffraction (2006). *CrysAlis CCD* and *CrysAlis RED* Oxford Diffraction, Abingdon, England.

[bb11] Pazicky, M., Loos, A., Ferreira, M. J., Serra, D., Vinokurov, N., Rominger, F., Jakel, C., Hashmi, A. S. K. & Limbach, M. (2010). *Organometallics*, **29**, 4448–4458.

[bb12] Raubenheimer, H. G., Lindeque, L. & Cronje, S. (1996). *J. Organomet. Chem.* **511**, 177–184.

[bb13] Sheldrick, G. M. (2008). *Acta Cryst.* A**64**, 112–122. 10.1107/S0108767307043930 18156677

[bb14] Teyssot, M., Jarrousse, A., Manin, M., Chevry, A., Roche, S., Norre, F., Beaudoin, C., Morel, L., Boyer, D., Mahioue, R. & Gautier, A. (2009). *Dalton Trans.* pp. 6894–6902. 10.1039/b906308k 20449127

[bb15] Wang, H. M. J., Vasam, C. S., Tsai, T. Y. R., Chen, S., Chang, A. H. H. & Lin, I. J. B. (2005). *Organometallics*, **24**, 486–493.

